# Functional estimation of bony segment lengths using magneto-inertial sensing: Application to the humerus

**DOI:** 10.1371/journal.pone.0203861

**Published:** 2018-09-12

**Authors:** Michele Crabolu, Danilo Pani, Luigi Raffo, Maurizio Conti, Andrea Cereatti

**Affiliations:** 1 Department of Electrical and Electronic Engineering, University of Cagliari, Cagliari, Italy; 2 Department of Clinical and Experimental Medicine, University of Sassari, Sassari, Italy; 3 Department POLCOMING, University of Sassari, Sassari, Italy; 4 Interuniversity Centre of Bioengineering of the Human Neuromusculoskeletal System, Sassari, Italy; 5 Department of Electronics and Telecommunications, Politecnico di Torino, Torino, Italy; Universite de Nantes, FRANCE

## Abstract

Inertial sensor technology has assumed an increasingly important role in the field of human motion analysis. However, the reliability of the kinematic estimates could still be critical for specific applications in the field of functional evaluation and motor rehabilitation. Within this context, the definition of subject-specific multi-body kinematic models is crucial since it affects the accuracy and repeatability of movement reconstruction. A key step for kinematic model calibration is the determination of bony segment lengths. This study proposes a functional approach for the in vivo estimation of the humerus length using a single magneto-inertial measurement unit (MIMU) positioned on the right distal posterior forearm. The humerus length was estimated as the distance between the shoulder elevation axis and the elbow flexion–extension axis. The calibration exercise involved five shoulder elevations in the sagittal plane with the elbow completely extended and five elbow flexion–extensions with the upper arm rigidly aligned to the trunk. Validation of the method was conducted on five healthy subjects using the humerus length computed from magnetic resonance imaging as the gold standard. The method showed mean absolute errors of 12 ± 9 mm, which were in the estimate of the humerus length. When using magneto-inertial technology, the proposed functional method represents a promising alternative to the regressive methods or manual measurements for performing kinematic model calibrations. Although the proposed methodology was validated for the estimation of the humerus length, the same approach can be potentially extended to other body segments.

## Introduction

In recent years, the use of magneto-inertial technology has assumed an increasingly important role in the field of human motion analysis. Despite the technological advances, the reliability of kinematic and kinetic quantities could still be critical for specific applications in the field of functional evaluation and rehabilitation. Within this context, the definition of personalised multi-body kinematic models is crucial to describe the movement in a reproducible and accurate manner [1–4]. A key step for kinematic model personalisation is the determination of bony segment lengths. This information is required for forward/inverse kinematic applications [5] and for the determination of the body segment inertial parameters [6].

Depending on the specific application, segment lengths can be measured or estimated in different ways [5,7–12]. A practical approach is to establish a mathematical relationship between the body segment lengths and stature and to compute the relevant ratios (*proportionality constants* reported in the anthropometric tables) [7]. Although several refinements of such constants were proposed in successive studies by increasing the number of subjects analysed, errors due to physiological and pathological anthropometric variability are unavoidable [10,13]. Conversely, when optoelectronic stereophotogrammetric systems are used, the segment lengths can be measured by placing skin markers on selected bony anatomical landmarks and then measuring the inter-marker distance [9]. The use of stereophotogrammetry, or other absolute position measurement systems, guarantees a considerably accurate inter-marker distance reconstruction, but it entails operator-dependent identification of the anatomical landmarks for the marker placement. The latter operation is associated with some errors, which may depend on the level of expertise of the operator and on the specific anatomical landmark being identified [14].

In contrast to stereophotogrammetry, magneto-inertial sensing technology does not supply reliable positional information. This means that when using magneto-inertial measurement units (MIMUs), the body segment lengths are determined using a measuring tape or estimated by *proportionality constants* [5].

Yuan et al. first attempted to automatically estimate the body segment lengths using MIMUs [15]. They estimated the lower limb lengths by optimizing the human body kinematic parameters when the posture was known. This was accomplished by using MIMUs attached on the limbs and controlling the end-effectors’ position by using footprint templates marked on the ground. However, the effectiveness of the proposed method was not quantified due to the lack of gold standard segment length measurements.

In a previous study [16], we presented a functional method for estimating the centre of rotation of the glenohumeral joint using an MIMU placed on the upper arm. Building upon such a milestone, in this study, we propose an original method that is based on a functional approach for the automatic estimation of a bony segment length using a single MIMU attached to the subject’s wrist. The method is applied to the estimate of the humerus length, and its accuracy was assessed using magnetic resonance imaging (MRI) as the gold standard.

## Material and methods

### Humerus length estimation

The proximal and distal ends of the humerus form the shoulder and elbow joints, respectively. The shoulder joint can be well modelled as a spherical joint [17,18], whereas the humeral–ulnar joint can be modelled as a hinge [19,20].

**Fig 1:**
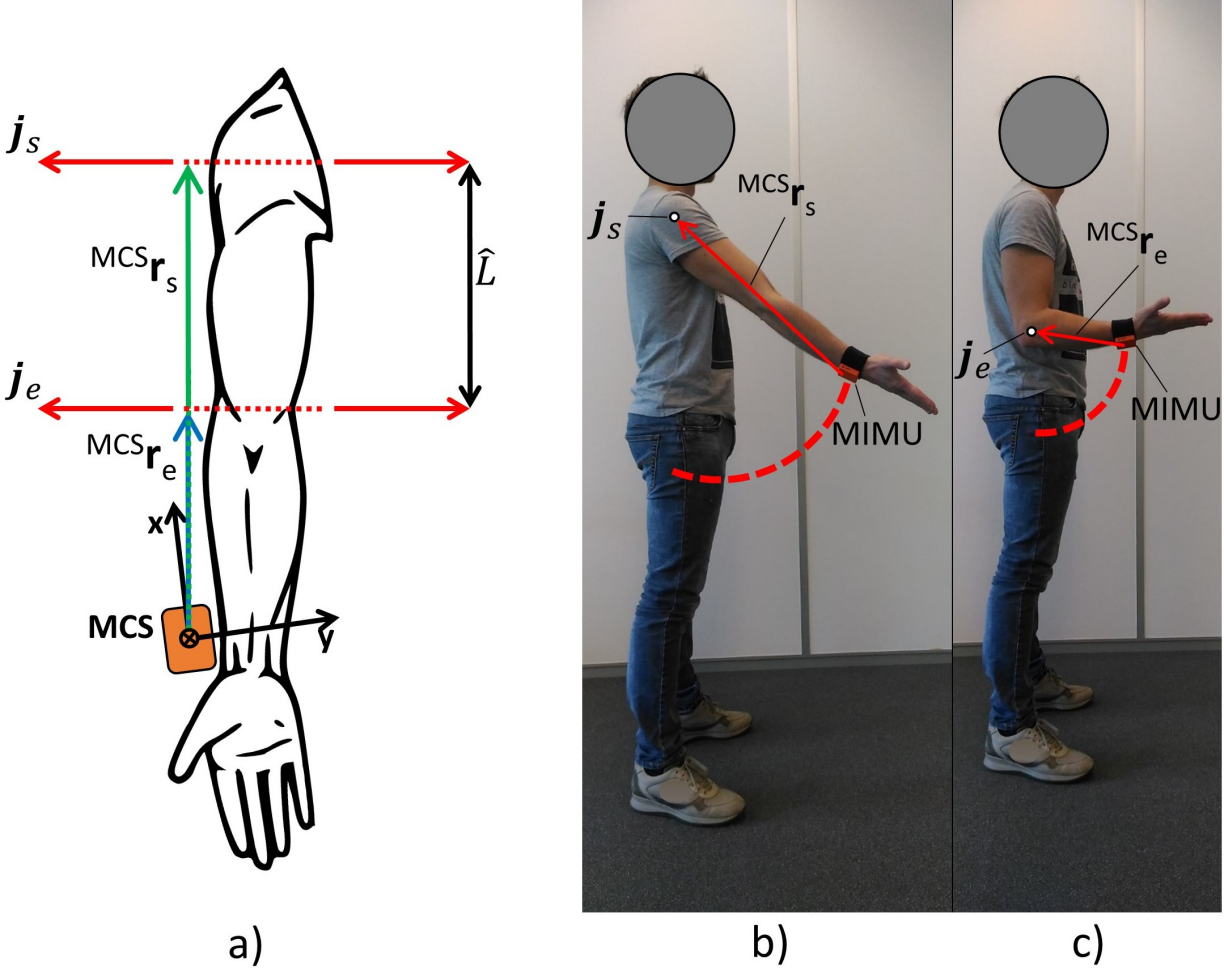
Experimental setup. a) The figure displays the axes of rotation (**j**_s_ and **j**_e_; in red), the radius vector with respect to the MIMU coordinate system (MCS) during a shoulder elevation in the sagittal plane (^MCS^**r**_s_; green arrow), the radius vector with respect to the MCS during an elbow flexion–extension (^MCS^**r**_e_; blue arrow), the estimated humerus length $\hat{L}$ (black double-ended arrow) and the MIMU (orange box) with its MCS (black arrows). b) Shoulder elevation in the sagittal plane. c) Elbow flexion–extension.

The proposed method estimates the humerus length *L* as the distance between the elbow and the shoulder flexion–extension rotation axes, as determined using the functional approach. In general, the functional approach records the movement data (i.e. marker trajectories with stereophotogrammetry systems or linear accelerations and angular velocities with MIMUs) while a subject performs an *ad-hoc* joint movement in order to determine the optimal centre of rotation (CoR) or the axis of rotation (AoR) [19,21,22]. In particular, let us consider an MIMU rigidly attached on the forearm (Fig 1).

During the shoulder elevation in the sagittal plane [23], the MIMU rotates about a quasi-stationary axis (**j**_s_), passing through the shoulder CoR, with a radius vector **r**_s_ pointing from the MIMU coordinate system (MCS) to **j**_s_. The modulus of **r**_s_ represents the distance between the origin of the MCS and **j**_s_. When an elbow flexion–extension movement is performed, the MIMU rotates about the quasi-stationary axis **j**_e_, passing approximately through the centre of the trochlea [24], with a radius vector **r**_e_ pointing from the origin of the MCS to **j**_e_. The humerus length *L* is then estimated as the modulus of the difference between **r**_s_ and **r**_e_. To effectively determine the humerus length, it is necessary that during the shoulder elevation, the elbow joint does not move and that both the shoulder and elbow movements are executed in the sagittal plane, avoiding any trunk swing (Fig 1). In a previous study [16], a method called Null Acceleration Point (NAP_ω_) was presented for the functional estimation of the shoulder CoR. Building on that result, herein, that method was used to estimate the radii **r**_s_ and **r**_e_. For a complete description of the algorithm that estimates the rotational radii, please refer to [16,25]. According to the NAP_ω_ algorithm, the acceleration **a** of the origin of the MCS fixed with the forearm during a pure rotational motion can be expressed as follows:

$$a=K(\omega ,\dot{\omega })r,$$ (1)

where **r** is the radius vector pointing from the MCS origin to the CoR and representing the CoR position, **ω** is the angular velocity, $\dot{\omega }$ is the angular acceleration and **K** assumes the following form [25]:

$$\left[\begin{array}{ccc} \left(-\omega_{y}^{2}-\omega_{z}^{2}\right) & \left(\omega_{x}\omega_{y}-{\dot{\omega }}_{z}\right) & \left({\dot{\omega }}_{y}+\omega_{x}\omega_{z}\right)\\ \left({\dot{\omega }}_{z}+\omega_{x}\omega_{y}\right) & \left(-\omega_{x}^{2}-\omega_{z}^{2}\right) & \left(\omega_{y}\omega_{z}-{\dot{\omega }}_{x}\right)\\ \left(\omega_{x}\omega_{z}-{\dot{\omega }}_{y}\right) & \left({\dot{\omega }}_{x}+\omega_{y}\omega_{z}\right) & \left(-\omega_{x}^{2}-\omega_{y}^{2}\right) \end{array}\right]$$ (2)

The quantities a, ω and $\dot{\omega }$ can be derived from the MIMU attached to the forearm. Applying Eq (1) at each N sampling time data recorded during the pure rotational motion of the forearm, a least-squares solution for **r** can be computed.

In a previous study [25], it was observed that slow calibration movements should be avoided since they lead to results that are worse than those obtained with fast movements, being the latter characterized by a higher signal-to-noise ratio (SNR) of angular velocity signals. Based on these considerations, to improve the SNR, the algorithm included in the least-squares solution computation only data samples characterised by a magnitude of ω larger than an empirically chosen threshold equal to 0.5 rad/s.

According to Eq (1), the NAP_ω_ algorithm can also be applied to estimate the distance between the MCS origin and a rotation axis. However, it is worth noting that if a perfect single-axial rotation occurs, the matrix **K** in Eq (1) would lose its full rank. For instance, if the axis of rotation is aligned with the x-axis of the MCS and no measurement noise is present, it can be observed that $\omega_{y}=\omega_{z}={\dot{\omega }}_{y}={\dot{\omega }}_{z}=0$, indicating that a unique solution does not exist. However, when real data are recorded, it is unlikely that the subject could perform a pure rotation about a unique AoR. This circumstance along with the presence of noise, intrinsic in the MIMU signals, guarantees that a solution for Eq (1) (i.e. the radius vector **r**) is found also when a flexion–extension joint movement is analysed.

In this study, we employed the NAP_ω_ algorithm to estimate the radius vectors **r**_s_ and **r**_e_ with respect to the MCS of an MIMU attached to the forearm during a shoulder elevation in the sagittal plane and elbow flexion–extension movements. The radius vectors are defined as follows:

$$r_{s}=K_{s}^{†}(\omega_{s},{\dot{\omega }}_{s})\;a_{s},$$ (3)

$$r_{e}=K_{e}^{†}(\omega_{e},{\dot{\omega }}_{e})\;a_{e},$$ (4)

where ***K***^**†**^ **= (*K***^***T***^***K*)**^**−1**^***K***^***T***^ is the pseudo-inverse of ***K***. The subscripts *s* and *e* indicate that the MIMU data were recorded during the shoulder sagittal elevation and elbow flexion–extension, respectively. The distance $\hat{L}$ was estimated as the Euclidean norm of the difference between the two radii **r**_**s**_ and **r**_**e**_:

$$\hat{L}=\|r_{s}-r_{e}\|.$$ (5)

**Fig 2:**
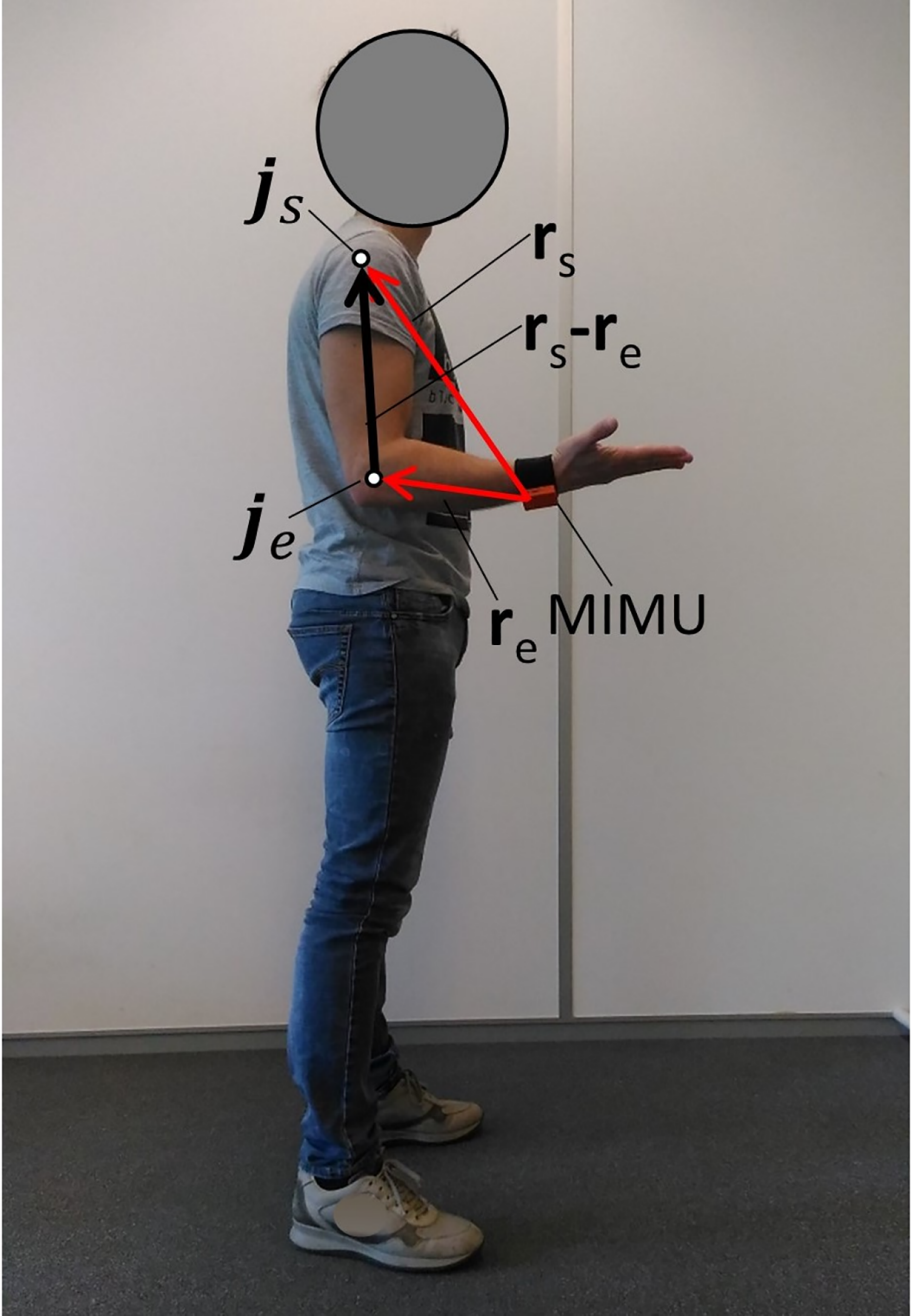
Extreme case in which the elbow joint is not extended during the shoulder motion. The vectors **r**_s_ and **r**_e_ and their vector difference when shoulder elevation is performed with the elbow in a non-extended configuration (i.e. at 90°).

The estimated humerus length $\hat{L}$ is computed as the modulus of the vector difference to guarantee the estimation is not affected by the case **r**_s_ and **r**_e_ do not have the same direction, for example when the elbow joint is not extended during the shoulder joint motion (Fig 2).

### Population study

Five healthy subjects [three males (M) and two females (F)] were enrolled in the study. The volunteers’ age, height and body mass index (BMI) were 36 ± 4 years, 1.7 ± 0.1 m and 20.7 ± 1.7 kg/m^2^, respectively. The study involved healthy doctoral students and academic staff from the bioengineering and medicine departments of the University of Sassari and the University of Cagliari, Italy. The study was performed by following the principles outlined in the Helsinki Declaration of 1975, later revised in 2000. All participants signed the informed consent for a standard MRI exam, approved by the ethics committee of the University of Sassari, before starting the recording. The protocol with MIMUs did not required the ethics committee approval since there were neither safety issues concerning their use nor clinical decisions taken on the protocol outcomes.

Each subject underwent an MRI examination and followed a protocol for the evaluation of the humerus length estimation.

### Determination of the humerus reference length from MRI

**Fig 3:**
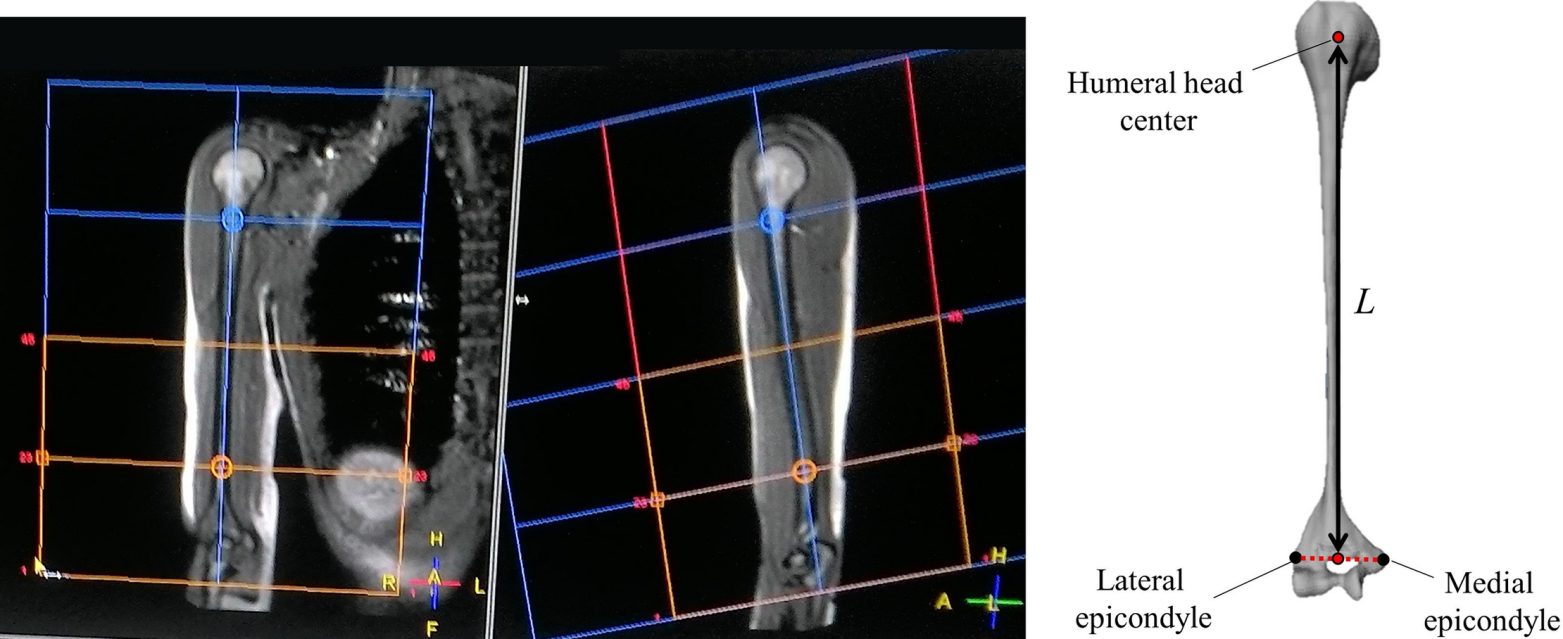
Magnetic resonance imaging (MRI)-based humerus length measurement. Two MRI slices on the frontal plane are shown on the left-hand side, and the 3D reconstruction of the humeral bone is shown on the right-hand side. The reference humerus length value *L* is obtained as the distance between the humeral head centre and the midpoint between the two distal epicondyles of the reconstructed humeral bone.

The MRI data of each subject’s right humerus were acquired. The MR scans of the whole right humerus were obtained using a 1.5-T MR scanner [Philips Intera Achieva (version 1.7)]. Spin-echo imaging sequences were used (axial T1-W: TR 660 ms, TE: 18 ms, flip angle: 90°, contiguous slice thickness: 4 mm and FoV: 280 mm). The 3D reconstructions of the entire humeral bone were obtained using the AMIRA image processing software package (version 5.4; Visualization Sciences Group) through a semiautomatic segmentation procedure, based on the first rough automatic segmentation, followed by a manual refinement of the bone contours on the slices that were incorrectly segmented [26]. The reference humerus length value *L* was computed as the distance between the humeral head centre and the midpoint between the lateral and medial distal epicondyles of the reconstructed humeral bone (Fig 3). The humeral head centre was estimated as the geometrical centre of the best fitting sphere to the humeral head of the reconstructed humeral bone. The level of precision associated with the identification of the humeral head centre was about 0.3 mm [27].

### Experimental protocol

A single MIMU was attached on the right distal posterior forearm through a Velcro strap, as shown in Fig 1 b–c. With the arm in the standard anatomical position, the MIMU was fixed with the x-axis approximately directed superiorly along the long axis of the forearm, the y-axis pointing laterally from the thumb to the little finger and the z-axis posteriorly. The MIMU comprised a triaxial accelerometer, a gyroscope and a magnetometer (Xsens Technologies BV, MTw2 Awinda wireless motion tracker system; sampling frequency = 100 Hz; dynamic accuracy: Roll/pitch = 0.75° RMS; Heading = 1.5° RMS). A 15-min warm-up period was included before starting the data collection. A preliminary spot check, 30 s in duration, of the MIMU orientation estimates was performed [28].

Subjects were instructed to perform the following calibration movements while minimising the translational motions of the interested joint: starting from the anatomical position, the subject was asked to perform five shoulder elevations in the sagittal plane S_EL_ with a range of motion (ROM) of approximately 60°, maintaining the elbow rigid and completely extended. Then, starting from the anatomical position, the subject was asked to execute five elbow flexion–extension (E_FL-EX_) movements with an ROM of approximately 90°, maintaining the upper arm rigidly aligned to the trunk. The ROM of the shoulder elevation in the sagittal plane was chosen to guarantee a sufficiently wide humeral rotation with a limited scapulohumeral rhythm and spinal tilt rotation [16,29,30]. Both the S_EL_ and E_FL-EX_ movements were performed maintaining the wrist rigid as at the starting configuration. Each subject repeated both S_EL_ and E_FL-EX_ movements three times for a total of six recordings per subject.

For a single subject, the humerus length *L* can be estimated through the following steps:

- Estimate the radius vector during S_EL_ with respect to the MCS (^MCS^**r**_s_) using the NAP_ω_ algorithm.
- Estimate the radius vector during an elbow flexion–extension (^MCS^**r**_e_) using the NAP_ω_ algorithm.
- Compute $\hat{L}$ as the modulus of the difference between the two radius vectors:

$$\hat{L}=\|r_{s}-r_{e}\|.$$ (6)

### Accuracy and precision assessment

For each subject, three independent estimates of both **r**_s_ and **r**_e_ were obtained, (three movements for S_EL_ and three E_FL-EX_ movements). Next, $\hat{L}$ was computed [Eq (6)], for each of the nine possible pairs of combinations of **r**_s_ and **r**_e_. For each combination, the error *e* between the reference humerus length *L* and the estimated $\hat{L}$ was calculated as follows:

$$e=L-\hat{L}.$$ (7)

For each subject, the accuracy and precision were computed as the mean absolute error (MAE) and standard deviation (SD):

$$MAE=\frac{1}{M}\sum_{i=1}^{M}\left|e_{i}\right|,$$ (8)

where *M* represents the combinations of the three pairs of **r**_s_ and **r**_e_. Furthermore, the grand mean (GM) and the SD amongst the subjects was computed. In addition, the average and SD of **r**_s_ and **r**_e_ amongst the three movements were calculated.

## Results

The average and SD values of the radius vectors, computed for the recorded movements (three S_FL-EX_ and three E_FL-EX_ movements), for each subject are listed in Table 1. The BMI and gender, the reference length values *L* estimated from the MRI and the estimated value $\hat{L}$ with the MAE and SD values are listed in Table 2. The GM and SD amongst the five subjects are also reported.

**Table 1:** Estimated average minimum rotational radii and standard deviation (SD) for each subject.

	Subject 1	Subject 2	Subject 3	Subject 4	Subject 5
${}^{MCS}{\bar{r}}_{sx}$ **± SD [mm]**	−482 ± 8	−460 ± 11	−441 ± 14	−404 ± 3	−492 ± 14
${}^{MCS}{\bar{r}}_{sy}$ **± SD [mm]**	23 ± 3	75 ± 3	-36 ± 5	34 ± 2	32 ± 6
${}^{MCS}{\bar{r}}_{sz}$ **± SD [mm]**	126 ± 3	88 ± 7	52 ± 3	44 ± 4	137 ± 7
${}^{MCS}{\bar{r}}_{ex}$ **± SD [mm]**	−207 ± 1	−197 ± 2	−157 ± 3	−184 ± 1	−221 ± 3
${}^{MCS}{\bar{r}}_{ey}$ **± SD [mm]**	−17 ± 4	−7 ± 2	−10 ± 2	−17 ± 1	1 ± 2
${}^{MCS}{\bar{r}}_{ez}$ **± SD [mm]**	51 ± 4	64 ± 3	47 ± 1	53 ± 6	52 ± 2

**Table 2:** Mean absolute error (MAE) ± SD for each subject and grand mean (GM) ± SD between the estimated humerus length $\hat{L}$ and the actual length *L* measured via magnetic resonance imaging (MRI). Body mass index (BMI; gender) of the subjects are also reported.

	**Subject 1**	**Subject 2**	**Subject 3**	**Subject 4**	**Subject 5**	**GM ± SD**
***BMI***	22.5 (M)	22.2 (M)	19.2 (F)	18.7 (F)	21.1 (M)	
***L* [mm]**	284.4	285	262.8	241.2	287.1	
$\hat{L}$ **[mm]**	288 ± 6	276 ± 9	285 ± 12	225 ± 3	286 ± 10	
***MAE* [mm]**	7 ± 3	9 ± 8	22 ± 12	16 ± 3	8 ± 5	12 ± 9

## Discussion

On average, the absolute errors associated with the humerus length estimation were equal to 12 ± 9 mm. There could be several explanations for the errors, one of which may be because the reference and estimated humerus length values were computed using two different approaches based on slightly different definitions of the humerus length. In fact, whereas the reference humerus length was measured from the MRI as the Euclidean distance between the geometrical centre of the humeral head and the midpoint between the two distal epicondyles, the humerus length estimated by the functional method was computed as the distance between the optimal elbow flexion–extension rotation axis and the optimal gleno-humeral flexion–extension rotation axis. Furthermore, improper execution of the functional movements (i.e. difficulty in keeping the trunk stationary and/or the elbow rigidly locked while moving the shoulder and vice versa) are expected to affect the estimation. In this regard, further studies are required for validating the proposed methodology on subjects with neuro-muscular and orthopaedic impairments. Another potential source of error was constituted by the presence of soft tissue artefacts, which may introduce relative movements between the body and the MIMU [31]. However, since the MIMU was positioned on the right distal posterior forearm close to the wrist and the wrist joint was kept firm during the recorded movements, the effects of the soft tissue artefact are expected to be a minor issue.

The NAP_ω_ algorithm assumes the stationarity of the AoR or CoR during the segment motion. In the shoulder [12, 24, 32] and the elbow [33], both CoR and AoR are not completely stationary with respect to the MCS. Furthermore, during the execution of both the shoulder and elbow movements, a limited sway of the trunk could possibly occur. To reduce the undesired motions of the joints due to trunk swaying, both the elbow and shoulder functional movements could be executed in a sitting position with the backrest supporting the back. To reduce the shoulder motion during the elbow flexion–extension (E_FL-EX_ movement), it could be useful to rest the proximal end of the ulna on a table [19].

The majority of the existing regressive methods provide estimates of the distance between external palpable anatomical landmarks based on the subject’s height [13,34]; therefore, a direct comparison with the results obtained by functionally estimating the segment length cannot be performed. However, it is worth mentioning that Fromuth et al. found that for a fixed subject height, the range of lengths for a specific body segment can exhibit large variations (>100 mm in the trochanter height) [13]. Gannon et al. [34] validated the anthropometric predictions derived from the *proportionality constants* of stature and reported a prediction accuracy lower than 25 mm for 74% of the estimated functional leg lengths. The regressive equation proposed by De Leva [8] allows to estimate the joint centres, and therefore the segment length, knowing the distance between the accessible landmarks; unfortunately, a quantitative validation was not reported.

Herein, the humerus length was estimated as the distance between the elbow and shoulder rotation axes; however, this is not the only option available when a functional approach is used. In fact, having performed non-planar movements, like star- or cross-shoulder motions [16], the position of the shoulder CoR could have been determined and the humerus length could have been estimated as the distance between the shoulder CoR and the elbow flexion–extension axis.

## Conclusion

In this study, a method to functionally estimate the humerus length in vivo using a single MIMU attached to the distal part of the forearm was presented and evaluated. The method’s accuracy was preliminary assessed for five healthy subjects using the humerus length computed from MRIs as the gold standard. When using magneto-inertial technology, the functional method proposed herein represents an alternative to the regressive methods or to time-consuming and costly measurement procedures for performing kinematic model calibration [15]. Although the proposed methodology was validated for the estimation of the humerus length, the same approach can be potentially extended to other body segments. However, due to the limited number of subjects analysed (five), caution is required when generalizing the study findings. The findings of this study encourage the application of this technique for the self-calibration of subject-specific multi-segment kinematic models. The future validation of the approach on subjects with movement impairments will reveal the applicability in such conditions, and consequently the potentialities for the use in rehabilitation and tele-rehabilitation contexts.

## Supporting information

S1 Data Data acquired during the experiment and employed in the data analysis. (7Z)
